# 3D model of harlequin ichthyosis reveals inflammatory therapeutic targets

**DOI:** 10.1172/JCI132987

**Published:** 2020-08-10

**Authors:** Florence Enjalbert, Priya Dewan, Matthew P. Caley, Eleri M. Jones, Mary A. Morse, David P. Kelsell, Anton J. Enright, Edel A. O’Toole

**Affiliations:** 1 Cell Biology and Cutaneous Research, Blizard Institute, Barts and the London School of Medicine and Dentistry, Queen Mary University of London, London, United Kingdom.; 2 Adaptive Immunity Research Unit, GlaxoSmithKline Medicine’s Research Centre, Stevenage, United Kingdom.; 3 Department of Pathology, University of Cambridge, Cambridge, United Kingdom.; 4 Centre for Inflammation and Therapeutic Innovation, Queen Mary University of London, London, United Kingdom.; 5 Department of Dermatology, Royal London Hospital, Barts Health NHS Trust ERN-Skin, London, United Kingdom.

**Keywords:** Dermatology, Genetics, Genetic diseases, Nitric oxide, Skin

## Abstract

The biology of harlequin ichthyosis (HI), a devastating skin disorder caused by loss-of-function mutations in the gene *ABCA12*, is poorly understood, and to date, no satisfactory treatment has been developed. We sought to investigate pathomechanisms of HI that could lead to the identification of new treatments for improving patients’ quality of life. In this study, RNA-Seq and functional assays were performed to define the effects of loss of *ABCA12* using HI patient skin samples and an engineered CRISPR/Cas9 ABCA12 KO cell line. The HI living skin equivalent (3D model) recapitulated the HI skin phenotype. The cytokines IL-36α and IL-36γ were upregulated in HI skin, whereas the innate immune inhibitor IL-37 was strongly downregulated. We also identified STAT1 and its downstream target inducible nitric oxide synthase (NOS2) as being upregulated in the in vitro HI 3D model and HI patient skin samples. Inhibition of NOS2 using the inhibitor 1400W or the JAK inhibitor tofacitinib dramatically improved the in vitro HI phenotype by restoring the lipid barrier in the HI 3D model. Our study has identified dysregulated pathways in HI skin that are feasible therapeutic targets.

## Graphical abstract

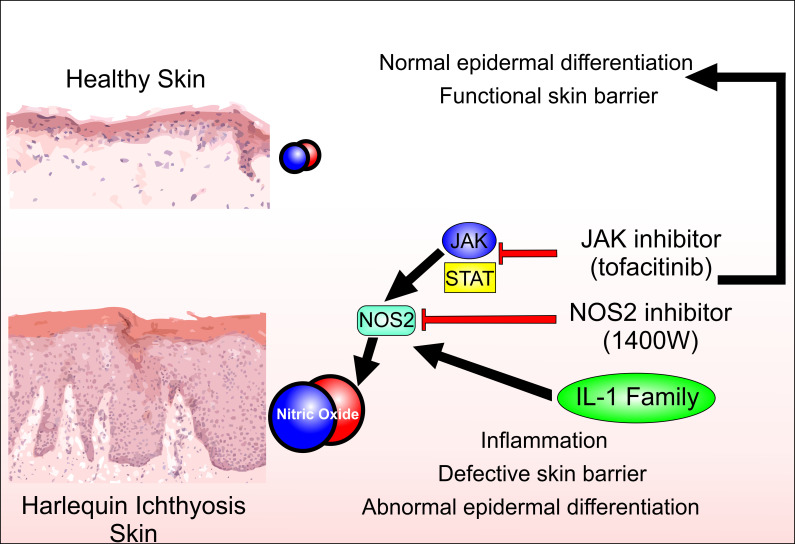

## Introduction

Harlequin ichthyosis (HI) (OMIM 242500) is the most severe and an often lethal form of the autosomal recessive congenital ichthyoses (1), a group of disorders with 2 other main clinical phenotypes: lamellar ichthyosis (LI) and nonbullous congenital ichthyosiform erythroderma (NBCIE) (2). The HI neonate presents at birth with a life-threatening skin phenotype, characterized by massively thickened skin with a markedly impaired skin barrier prone to infection and water loss, requiring intensive care treatment (3). Loss-of-function mutations in the lipid transporter ATP binding cassette A12 (*ABCA12*) gene are the cause of HI (4, 5). The ABCA12 transporter is important in delivering glucosylceramides (GluCer) to the lipid lamellae through lamellar bodies (LBs) (6). Current treatments of HI are daily topical application of emollients and, for severe cases, systemic therapy using oral retinoids (e.g., acitretin) (3). However, long-term retinoid treatment is associated with acute and chronic toxicities (7).

Since the discovery of *ABCA12* as the gene responsible for the HI phenotype, several mouse ABCA12 KO models have been developed and characterized (6, 8–10) showing good recapitulation of the human HI phenotype: hyperkeratosis, LB malformations, severe barrier dysfunction, and defective lipid homeostasis. These in vivo HI mouse models have limitations, as the mice die shortly after birth due to dehydration.

Skin inflammation is well known in HI from clinical observations (11); however, there are a limited number of studies investigating inflammatory dysregulation in HI. A proinflammatory signature was reported in the HI mouse embryo, suggesting a role for inflammation in HI pathogenesis (12). An increase of general inflammatory (IL-2), innate (IL-1β), IFN-γ, and IL-17–related cytokines (including *IL36G*) was reported in a heterogeneous group of ichthyosis patients (cohort excluding HI patients) (13); this was similar to the inflammatory response seen in psoriasis (13).

To study further the role of ABCA12 in human epidermis, Thomas et al. developed an in vitro HI 3D model using keratinocytes with retroviral shRNA targeting of *ABCA12* (14). This model recapitulated the HI-impaired epidermal differentiation phenotype: late differentiation markers, such as involucrin, were expressed in the epidermal suprabasal layer, correlating with HI in vivo observations (14). In this model, a moderate ichthyosis phenotype was observed, likely due to residual ABCA12 protein expression.

In this study, we developed an HI 3D skin model using a CRISPR/Cas9 ABCA12 KO cell line generated in-house, leading to a complete loss of ABCA12 protein. The model recapitulated HI patient skin features with altered keratinocyte differentiation and impaired lipid barrier formation. The RNA-Seq data analysis from 4 HI patient skin samples and the HI 3D model revealed dysregulation of epidermal development and lipid metabolism as well as inflammation, including the STAT pathway. We confirmed upregulation of the STAT1/inducible nitric oxide synthase (NOS2) signaling pathway in the HI 3D model and HI patient samples and showed that inhibition of NOS2 and STAT signaling resulted in improved skin-barrier formation in vitro.

## Results

### Alterations in intracellular lipid distribution, cell morphology, and proliferation in 2D cultured ABCA12 KO cells.

**Figure 1:**
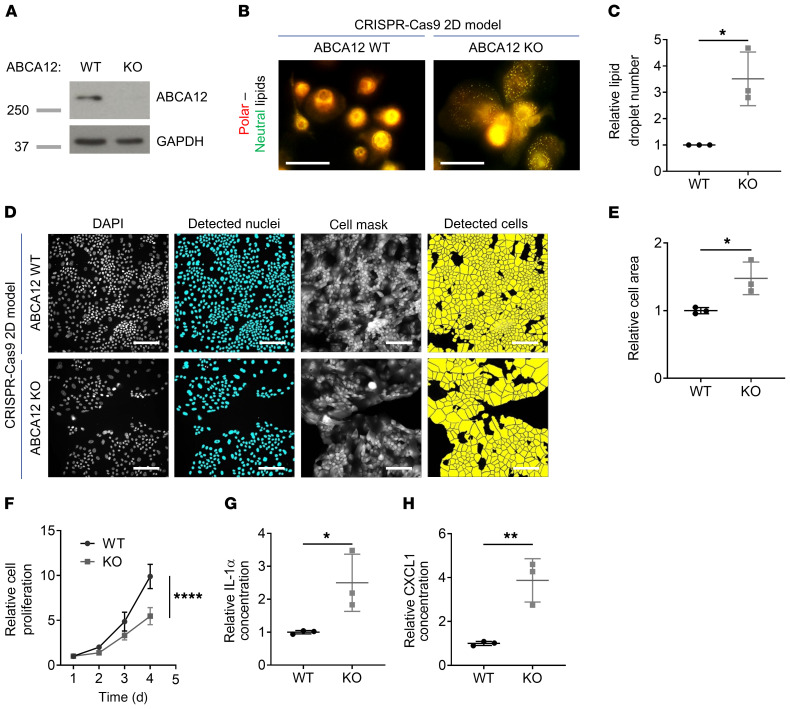
ABCA12 KO–induced changes in lipid distribution, cellular morphology and growth, and increased inflammatory response in 2D culture. (**A**) Representative immunoblot of ABCA12 and GAPDH proteins in ABCA12 WT and KO cell lysates. (**B**) Representative Nile red–staining images of polar/neutral (red/green channel) lipids in ABCA12 WT and KO cells. Scale bars: 50 μm. (**C**) Associated quantitative lipid droplet number analysis. Each dot represents the mean of 3 technical replicates. *n* = 3. Data are represented as mean ± SD. *P ≤ 0.05, unpaired *t* test. (**D**) Representative fluorescence staining images of CellMask and DAPI in ABCA12 WT and KO cells. Scale bars: 100 μm. (**E**) Associated quantitative cell area analysis. Each dot represents the mean of 3 technical replicates. *n* = 3. Data are represented as mean ± SD. *P ≤ 0.05, unpaired *t* test. (**F**) Cell proliferation analysis of ABCA12 WT and KO cells. *n* = 3. Data are represented as mean ± SD. ****P ≤ 0.0001, 2-way ANOVA with Šidák’s multiple comparisons test. Measurement of secreted (**G**) IL-1α and (**H**) CXCL1 in ABCA12 WT and KO supernatants. Each dot represents the mean of 3 technical replicates. *n* = 3. Mean ± SD. *P ≤ 0.05; **P < 0.01, unpaired *t* test.

We developed CRISPR/Cas9 ABCA12 WT and KO keratinocyte cell lines using the telomerase-immortalized N/TERT cell line, which has been validated as a biologically relevant substitute for human keratinocytes in 3D human epidermal models, with formation of a functional skin barrier, and also in inflammatory skin models (15, 16). The KO cell line bears a homozygous 2 bp deletion in *ABCA12* exon 27 (c.3832_3833delAC), resulting in a predicted frameshift with the production of 27 different amino acids after the mutation site followed by a premature stop codon: p.Thr1278Ilefs*28 (Supplemental Figure 1, A and B; supplemental material available online with this article; https://doi.org/10.1172/JCI132987DS1). In silico analysis showed no off-target mutations in the CRISPR/Cas9 ABCA12 KO cell line (Supplemental Figure 1C). Loss of ABCA12 protein expression was confirmed (Figure 1A) and resulted in major differences between the ABCA12 WT and KO 2D cultures. Altered lipid distribution characterized by a significant increase (3.5-fold) in the number of intracellular lipid droplets, a characteristic of HI stratum corneum keratinocytes (5, 17), was noticed in ABCA12 KO cells (Figure 1, B and C) with the use of Nile red staining detecting both polar and neutral lipids. Morphological changes were noted in ABCA12 KO cells using CellMask staining (Figure 1D): a significant 1.5-fold increase of cell area was identified in the ABCA12 KO compared with ABCA12 WT cells (Figure 1E), suggesting increased differentiation. The ABCA12 KO cell line displayed a decrease in cell proliferation, as determined by a significant reduction (1.8-fold) in the number of cells after 5 days of 2D culture (Figure 1, D and F). The levels of innate immune cytokines were assessed in the supernatant of the ABCA12 WT and KO cell lines using a human cytokine array; an increase was seen in several cytokines: CXCL1, IFN-γ, IL-1α, IL-1RA, IL-8, and IL-18. The significant increased secretion of CXCL1 and IL-1α was confirmed by ELISA (Figure 1, G and H, and Supplemental Figure 2).

### Dysregulation of keratinocyte differentiation, lipid expression, and increased inflammation in the HI 3D model replicating human HI adult skin.

**Figure 2:**
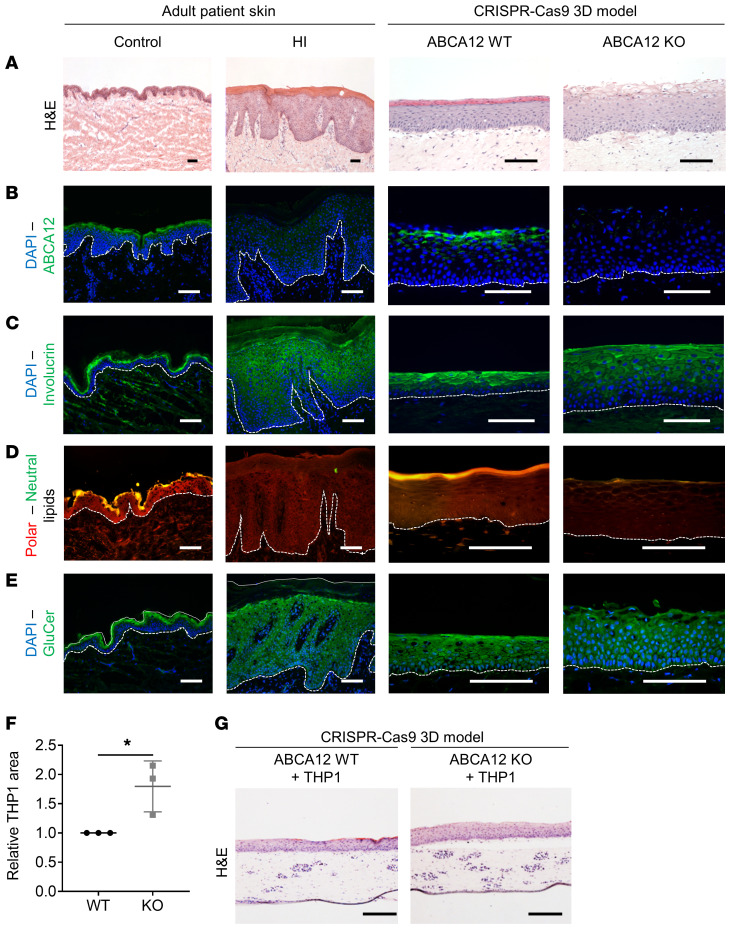
HI skin ABCA12 KO 3D model showed alterations in keratinocyte differentiation, lipid expression pattern, and inflammation. Representative (**A**) H&E (bright-field channel) staining images and (**B**) ABCA12 (green channel), (**C**) involucrin (green channel), (**D**) polar/neutral (red/green channel) lipid, (**E**) GluCer (green channel), and DAPI (blue channel) immunofluorescence staining images of control skin, HI patient skin, and in vitro WT and HI 3D models. Scale bars: 100 μm. (**F**) Quantitative analysis of relative THP-1 cellular area in the dermis-like layers of ABCA12 WT and KO cells. Each dot represents the mean of relative THP-1 area from 3 independent images. *n* = 3. Data are represented as mean ± SD. *P ≤ 0.05, unpaired *t* test. (**G**) Associated H&E-stained (bright-field channel) representative images. Scale bars: 200 μm.

To investigate the dysregulated intracellular signaling pathways following loss of ABCA12, we built an in vitro HI 3D model using the in-house–developed ABCA12 KO cell line and compared its phenotype to that of a WT 3D model and to 3 adult normal and HI patient skin samples. HI skin displayed abnormal keratinocyte differentiation, characterized by hyperkeratosis, acanthosis, and impaired and extended differentiation as well as defective lipid transport compared with adult normal control skin (Figure 2, A–E). We noted the presence of enucleated cells in suprabasal layers of HI skin, but variable differentiation in the abnormal HI stratum corneum (Figure 2A and Supplemental Figure 4, D–F). In 3D culture, the suppression of ABCA12 protein expression, confirmed by ABCA12 immunofluorescence and Western blot (Figure 2B and Supplemental Figure 3C), resulted in striking changes in the HI 3D model compared with the control. H&E staining revealed abnormal differentiation of the in vitro HI 3D model: as seen in HI skin (Supplemental Figure 4, D–F), enucleated cells were also observed in the spinous layer of the HI 3D model (Figure 2A). In the upper layer of the HI 3D model, undifferentiated large cells, some still nucleated, displayed variable expression of the late differentiation marker involucrin (Figure 2, A and C). Nile red staining analysis revealed the absence of an outermost lipid (polar and neutral) enriched layer in the in vitro HI 3D model (Figure 2D). GluCer staining was decreased in the aberrantly differentiated upper epidermal layers of the HI 3D model (Figure 2E). Human monocytic THP-1 cells added to the dermis-like layer of the 3D model displayed increased proliferation in the HI 3D model compared with the WT, as determined by quantification of the dermal area occupied by THP-1 cells suggesting a proinflammatory effect of loss of epidermal ABCA12 (Figure 2, F and G).

### Keratinocyte transcriptome in HI patient skin and the HI 3D model.

**Figure 3:**
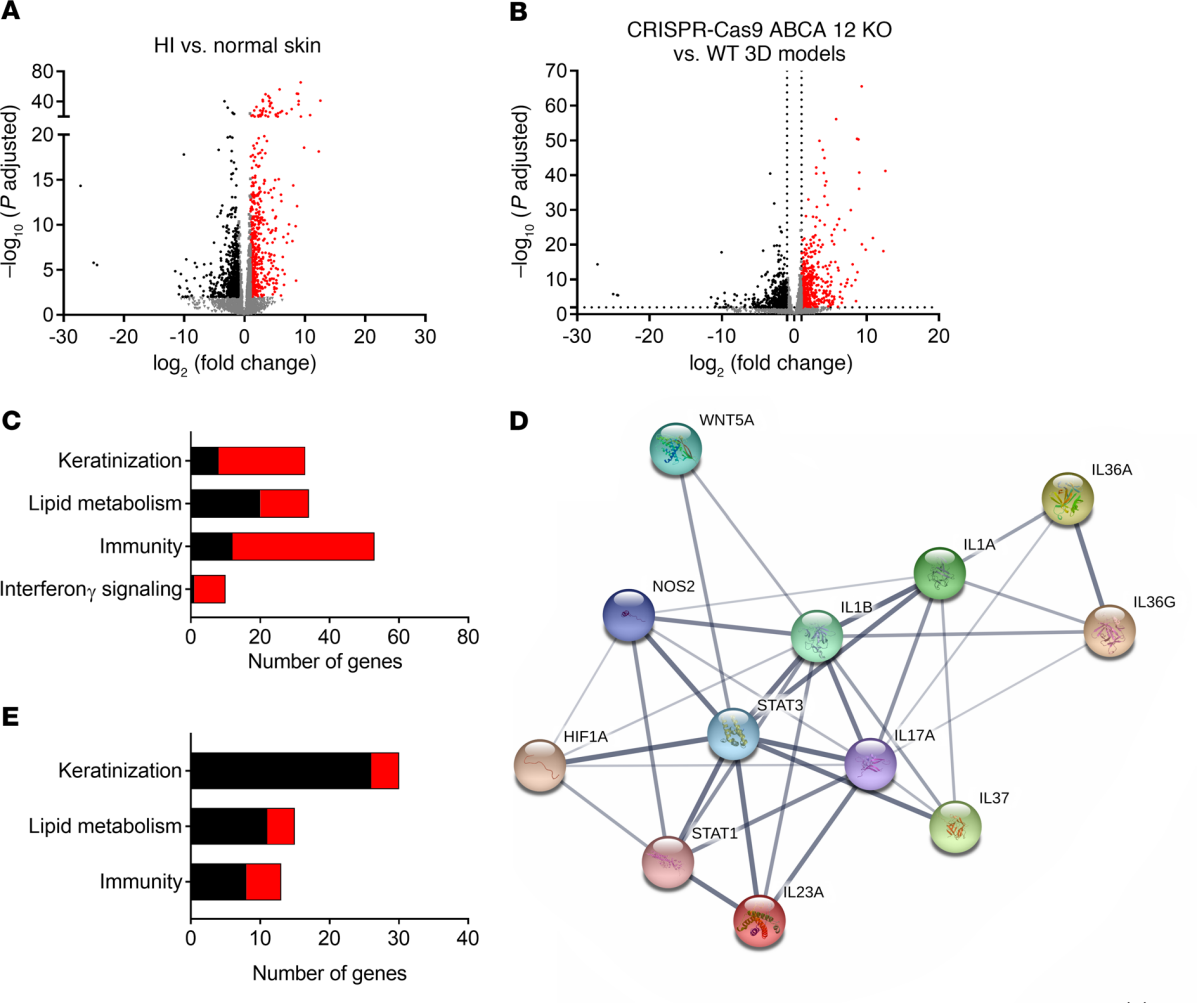
Transcriptomic profile of HI skin and ABCA12 KO model using RNA-Seq. Volcano plot of differentially expressed genes between (**A**) 4 HI skin and 5 normal skin controls and (**B**) CRISPR/Cas9 ABCA12 KO and WT 3D models. Each red/black dot represents a significantly differentially up- or downregulated gene. (**C**) GO terms enrichment in differentially expressed genes (upregulated in red, downregulated in black) in HI skin compared with normal skin. (**D**) Functional protein association network; line thickness indicates the strength of data support. (**E**) GO terms enrichment in differentially expressed genes (upregulated in red, downregulated in black) in CRISPR/Cas9 ABCA12 KO 3D models compared with control.

To examine the pathways modulated by ABCA12 protein deficiency and validate the relevance of the HI 3D model, RNA-Seq analysis was performed on normal and HI patient skin as well as WT and ABCA12 KO 3D models. RNA-Seq analysis revealed 714 upregulated and 824 downregulated genes in HI skin compared with control normal skin (Figure 3A). The Gene Ontology (GO) term enrichment analysis of the differential expressed genes (Figure 3C and Table 1) revealed that 32 were involved in epidermal development, including significant upregulation of keratins (e.g., *KRT16*) and markers of differentiation (e.g., *TGM1*). Thirty-four genes involved in lipid metabolism, such as *ALOX12B*, were differentially expressed. There were 53 differentially expressed genes in HI skin associated with immunity, mostly upregulated, for example, *IL36A* and *IL36G* (940.8- and 37.7-fold increase, adjusted *P* value [*P_adj_*] = 2.8 × 10^–19^ and 8.4 × 10^–22^, respectively), and strong downregulation of *IL37* (fold change 0.11, *P_adj_* = 4.7 × 10^–4^), an inhibitor of innate immunity, was seen. Additionally, *IL17* mRNA was upregulated (32.3-fold increase, *P_adj_* = 0.012). Also, 9 of 10 identified genes involved in IFN-γ signaling, such as *STAT1* (2.7-fold increase, *P_adj_* = 1.4 × 10^–3^), were significantly upregulated in HI skin compared with normal skin controls. Using STRING, connections between key genes involved in inflammation differentially expressed in the HI RNA-Seq data set, including STAT family members and the potential downstream target gene *NOS2*, were visualized (Figure 3D). Analysis of the RNA-Seq data using CIBERSORT identified an increased proportion of activated dendritic cells in HI skin, consistent with the very severe barrier defect (Supplemental Figure 5). In the ABCA12 KO 3D model RNA-Seq data set, 402 genes were significantly dysregulated (Figure 3B), clustering in 3 main GO terms, keratinization, lipid metabolism, and inflammation (Figure 3E and Table 2), which correspond to the same clusters as those identified in the HI skin data set (Figure 3C). In the keratinization cluster, there were 30 genes, mostly downregulated, such as *ABCA12* (fold change = 0.156, *P_adj_* = 3.08 × 10^–26^), loricrin (*LOR*) and several late cornification envelope (LCE) genes: *LCE1A*, *LCE1B*, *LCE1C*, *LCE1D*, *LCE1F*, *LCE2A*, *LCE2B*, *LCE2C*, *LCE2D*, *LCE3C*, *LCE3D*, *LCE3E*, *LCE4A*, *LCE5A*, *LCE6A*. Also 4 kallikrein genes, *KLK6*, *KLK7*, *KLK13*, and *KLK14*, were substantially downregulated. Additionally, 15 genes involved in lipid metabolism were differentially expressed, some directly involved in ichthyosis, e.g., *ABCA12*, *ALOX5*, *ALOX12B*, *ALOXE3*, *CYP4F2*, and 2 elongation of very long chain fatty acid protein (*ELOV*) genes. Moreover, 13 genes connected to cytokine activity were modulated, for example, the inhibitor of innate immunity *IL37* (fold change = 0.029, *P_adj_* = 2.4 × 10^–09^), the IL-36 receptor antagonist *IL36RN* (fold change = 0.42, *P_adj_* = 3.1 × 10^–05^), *IL24* (fold change = 0.28, *P_adj_* = 7.8 × 10^–05^), and the IL-1 family member *IL1F10*.

### Decreased IL-37 expression and increased NOS2 signaling in the in vitro HI 3D model.

**Figure 4:**
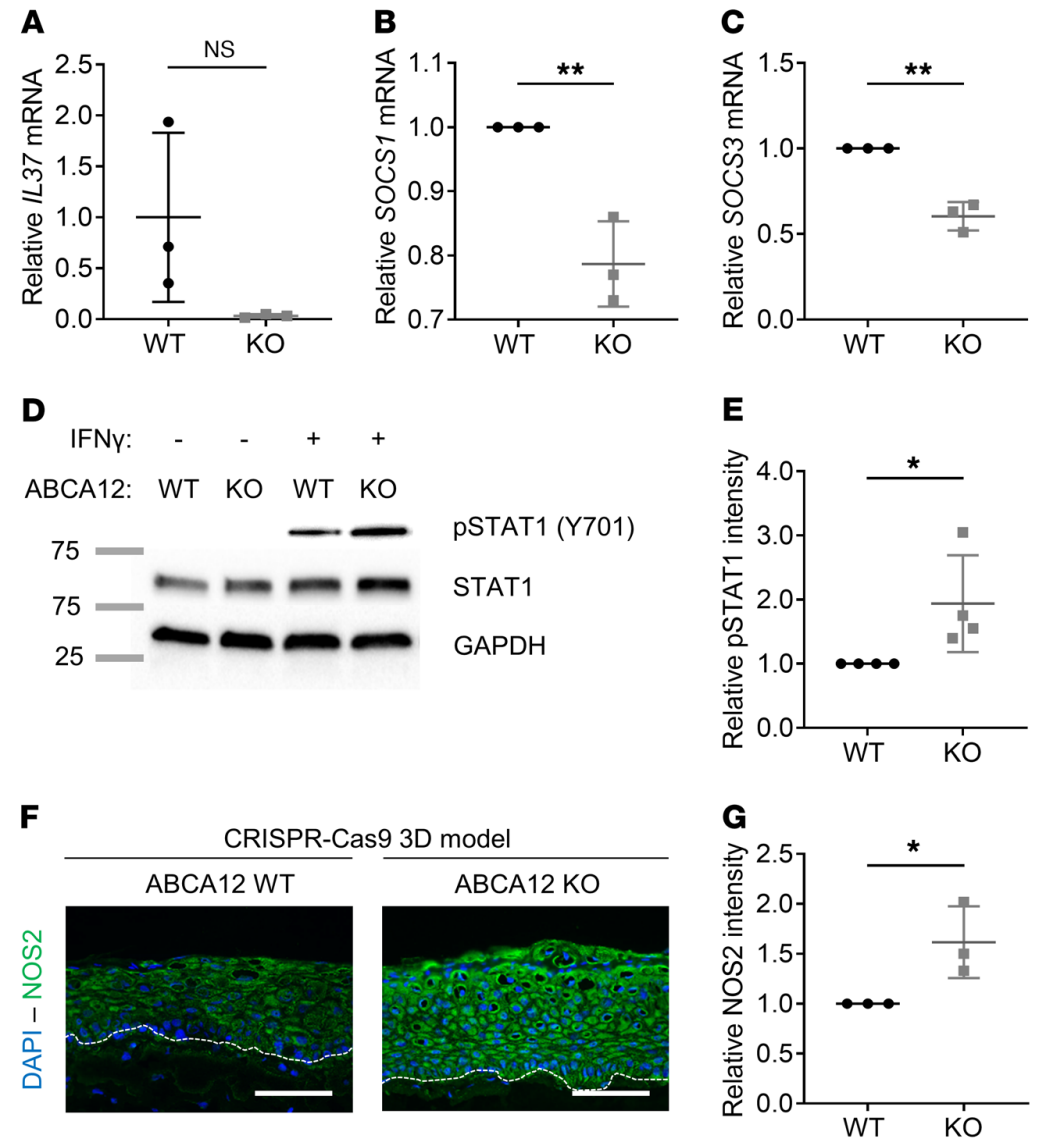
Decrease in antiinflammatory response and activation of NOS2 pathway in the in vitro ABCA12 KO 3D model. (**A**) qPCR analysis of *IL37* in ABCA12 WT and KO 3D models. *n* = 3. Data are represented as mean ± SD. NS, *P* = 0.068, unpaired *t* test. qPCR analysis of (**B**) *SOCS1* and (**C**) *SOCS3* in ABCA12 WT and KO 2D model cell lysates. Each dot represents the mean of 3 technical replicates. *n* = 3. Data are represented as mean ± SD. **P ≤ 0.01, unpaired *t* test. (**D**) Representative immunoblot of p-STAT1 (Y701), total STAT1, and GAPDH proteins in untreated (–) or stimulated (+) with IFN-γ ABCA12 WT and KO cell lysates and (**E**) associated p-STAT1 quantitative analysis. *n* = 4. Data are represented as mean ± SD. *P ≤ 0.05, unpaired *t* test. The p-STAT1 blot was run in parallel, contemporaneously, with total STAT1 and GAPDH blots. (**F**) Representative NOS2 (green channel) and DAPI (blue channel) staining images of in vitro WT and HI 3D models, and (**G**) associated quantitative NOS2 analysis. Each dot represents the mean of relative NOS2 intensity from 3 independent images. Scale bars: 100 μm. *n* = 3. Data are represented as mean ± SD. *P ≤ 0.05, unpaired *t* test.

In the HI 3D model, we first investigated the mRNA expression of IL-1 family cytokines, which were dysregulated in the HI skin RNA-Seq data set (Figure 3). We found *IL37* mRNA was strikingly decreased (Figure 4A), but no differences in *IL36* mRNA levels were observed (data not shown). Then, to follow up the RNA-Seq findings of IFN-γ–triggered signaling and the potential regulation of *NOS2* expression (Figure 3), quantitative PCR (qPCR) mRNA expression analysis of *SOCS1* and *SOCS3*, 2 inhibitors of the JAK/STAT pathway, was initially performed; this showed a significant decrease (Figure 4, B and C) in ABCA12 KO cells compared with WT. No phosphorylated STAT1 (p-STAT1) was detected in the in vitro HI 3D model; however, immunoblotting analysis showed a significant increase of p-STAT1 (Y701) in ABCA12 KO cells compared with WT following IFN-γ treatment (Figure 4, D and E). Also, a significant increase in the AP-1 subunit, FOSL2, was identified in the HI 3D model (Supplemental Figure 3C). The mRNA and protein expression of the STAT1 downstream target, NOS2, was significantly increased in the HI 3D model compared with the WT control (Supplemental Figure 3B and Figure 4, F and G).

### Upregulation of proinflammatory cytokines, STAT1, and NOS2 signaling in HI patient skin.

**Figure 5:**
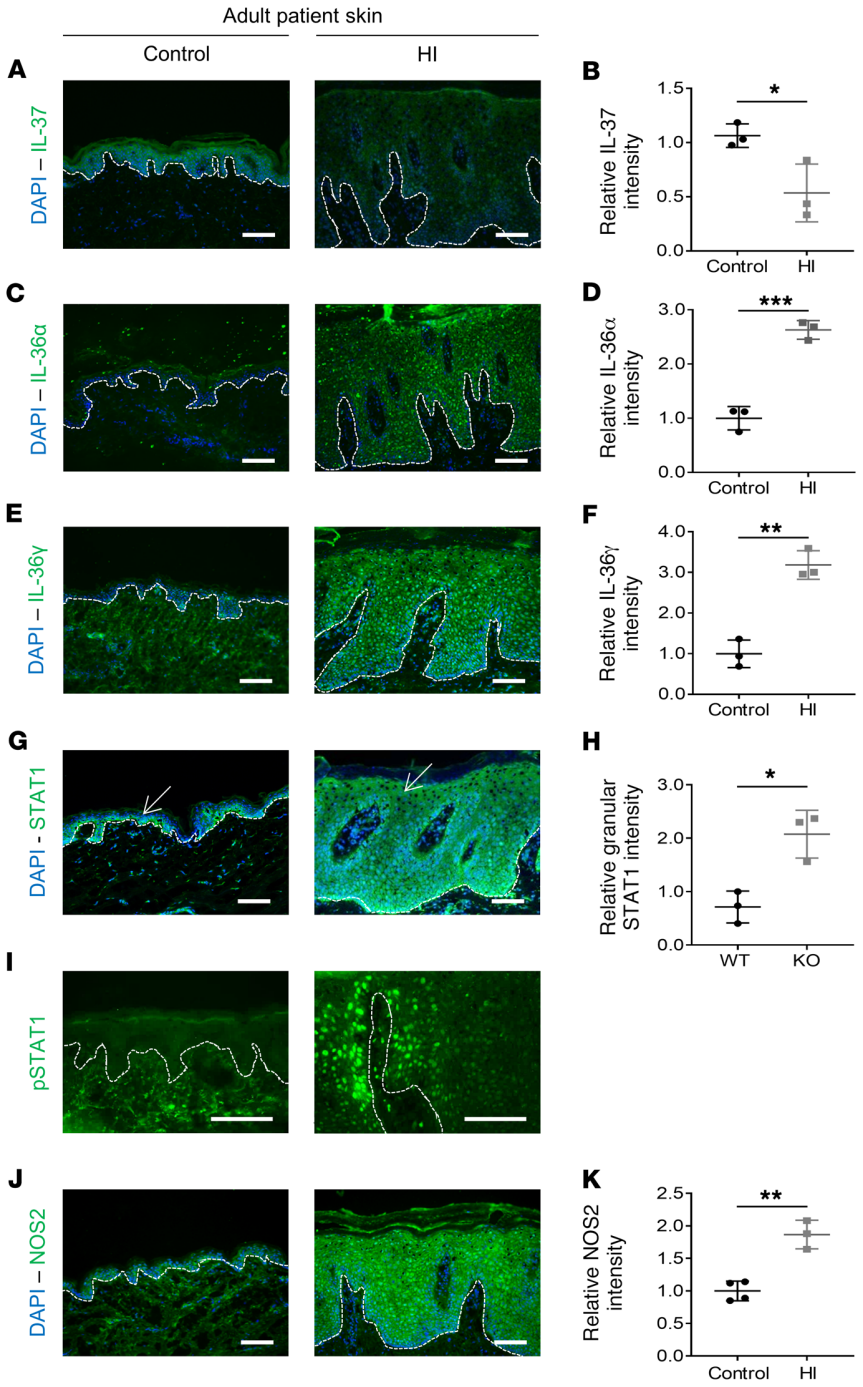
Inflammation and activation of the STAT1/NOS2 pathway in HI skin. Representative (**A**) IL-37, (**C**) IL-36α, (**E**) IL-36γ, (**G**) STAT1, (**I**) p-STAT1, (**J**) and NOS2 (green channel) and DAPI (blue channel) staining images of control skin and HI patient skin. Arrows indicate granular layer. Associated quantitative analysis of (**B**) IL-37, (**D**) IL-36α, (**F**) IL-36γ, (**H**) granular layer STAT1, and (**K**) NOS2 protein expression in control skin and HI patient skin. Each dot represents the mean of relative protein intensity from 3 independent images. *n* = 3 or 4. Data are represented as mean ± SD. *P ≤ 0.05; **P ≤ 0.01; ***P ≤ 0.001, unpaired *t* test. Scale bars: 100 μm.

To confirm the in vivo relevance of the dysregulation of IL-1 family cytokines and IFN-γ pathways, 3 adult HI skin patient samples, HI 1, 2, and 3, were examined further. We determined *IL37*, *IL36A*, and *IL36G* mRNA levels by qPCR (Supplemental Figure 4, I–K) and protein expression by immunofluorescence staining (Figure 5, A–F). Expression of these cytokines recapitulated the RNA-Seq results: IL-37 mRNA and protein expression were decreased in HI skin samples compared with normal skin controls (Figure 5, A and B, and Supplemental Figure 4I), whereas HI keratinocytes showed a significant increase in IL-36α and IL-36γ expression, both minimally expressed in control skin (Figure 5, C–F, and Supplemental Figure 4, J and K). In both cases, IL-36 expression was mainly cytoplasmic, but nuclear IL-36 was also detected. We also observed an alteration in total STAT1 expression pattern, with strong expression throughout HI adult epidermis, but restriction to the basal layer in normal skin by immunofluorescence analysis (Figure 5G and Supplemental Figure 4G). Nuclear total STAT1, suggesting phosphorylation, was also observed in the basal and suprabasal layers of HI skin. Quantification indicated a significant upregulation of total STAT1 protein expression in the granular layer of HI skin compared with normal skin (Figure 5H). Nuclear p-STAT1 (Y701), absent in normal epidermis, was also detected in HI epidermis (Figure 5I). STAT1 and p-STAT1 (Y701) were also found to be significantly upregulated by Western blotting in HPV-16 immortalized keratinocytes from an HI patient (HI 3) compared with expression in the WT control cell line (Supplemental Figure 4H). Finally, immunofluorescence analysis of NOS2 in HI skin demonstrated a significantly higher expression than the low level detected in normal skin (Figure 5, J and K).

### Modulation of NO in the HI 3D model reverses the phenotype.

**Figure 6:**
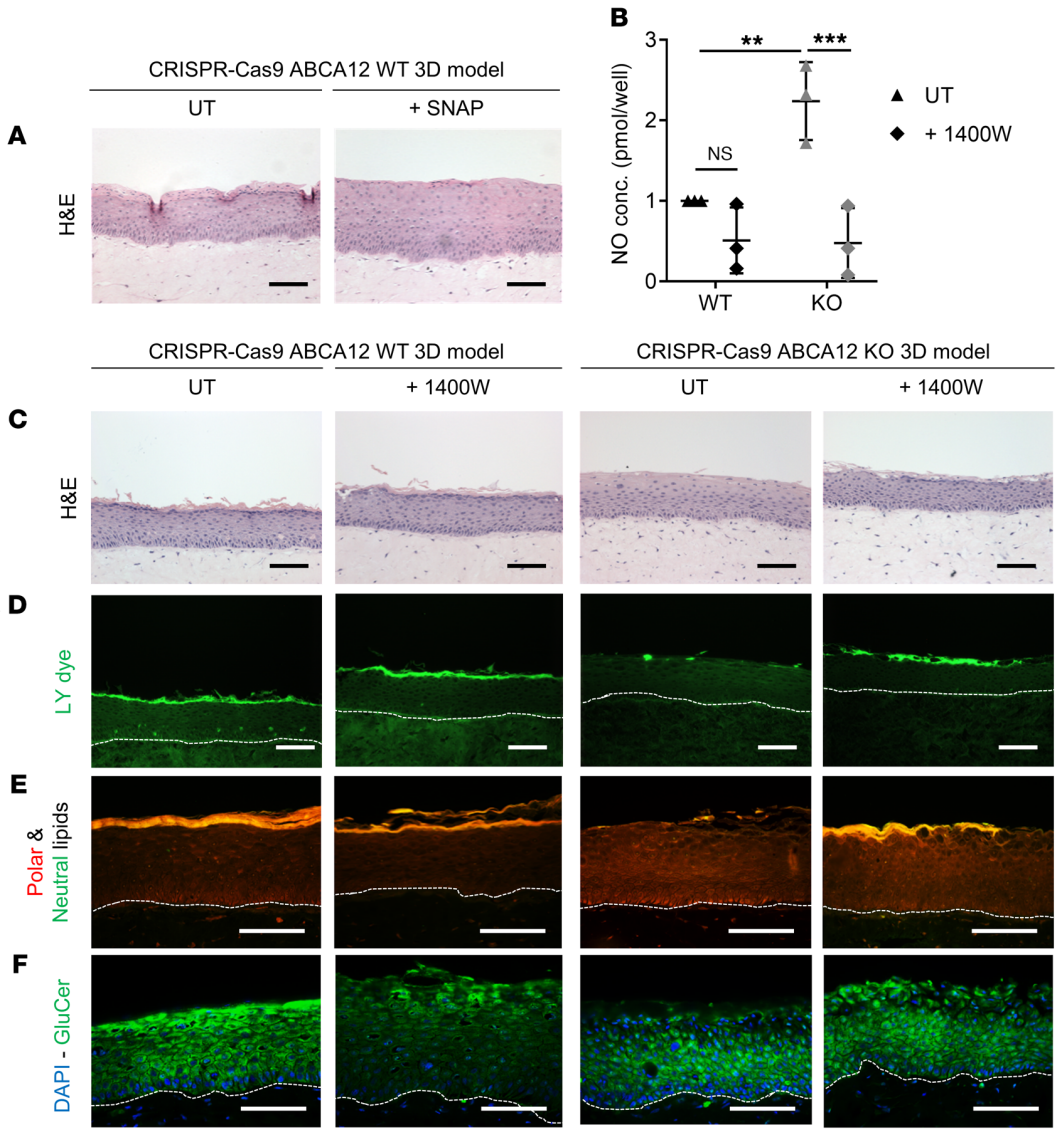
NO release caused epidermal acanthosis and inhibition of NOS2 resulted in normalization of the skin barrier in the HI 3D model. (**A**) Representative H&E (bright-field channel) images of in vitro WT and HI 3D models untreated (UT) or treated with SNAP compound. (**B**) Quantitative analysis of intracellular NO in in vitro WT and HI 3D models, with or without 1400 W inhibitor. Each dot represents the mean of 3 technical replicates. *n* = 3. Data are represented as mean ± SD. **P ≤ 0.01; ***P ≤ 0.001, 2-way ANOVA with Tukey’s multiple comparisons test. Representative (**C**) H&E (bright-field channel), (**D**) Lucifer yellow (LY) (green channel), (**E**) polar/neutral (red/green channel), (**F**) GluCer (green channel), and DAPI (blue channel) staining images of in vitro WT and HI 3D models from 3 independent biological replicates. Scale bars: 100 μm.

To test whether the identified upregulation of NOS2 in HI was involved in the HI-impaired barrier, the in vitro WT 3D model was treated with the NO-releasing agent SNAP. Treatment with SNAP induced acanthosis of the epidermis of the WT 3D model, as shown by H&E staining (Figure 6A).Moreover, intracellular NO levels were significantly higher in the HI 3D model (2.3-fold increase) compared with the WT (Figure 6B). To investigate further the role of NO in the abnormal differentiation in HI skin, WT and HI 3D models were treated with the selective NOS2 inhibitor 1400W. Treatment with 1400W significantly reduced the intracellular NO level in the 3D model, as determined by the quantification of total intracellular NO (Figure 6B), and strikingly improved the in vitro HI phenotype, whereas no significant differences were seen in the treated WT 3D model (Figure 6, C–F). H&E staining revealed the formation of a stratum corneum–like layer in the treated HI 3D model (Figure 6C). Reduction of Lucifer yellow dye penetration, a readout of impaired barrier, was also noted in the treated HI 3D model compared with the untreated control (Figure 6D). Inhibition of NOS2 in the HI 3D model resulted in restoration of the polar and neutral lipid–enriched outermost layer, determined using Nile red staining (Figure 6E). Moreover, GluCer was detected in the outermost layer of the in vitro HI 3D model treated with 1400W and was absent in the untreated HI 3D model, demonstrating reversion of a specific defect of HI epidermis by inhibition of NOS2 (Figure 6F). From the RNA-Seq data, we chose 2 transcripts important in differentiation and innate immunity that were altered in the ABCA12 KO 3D model data set compared with control. Extraction of mRNA from the 3D models treated with 1400W and qPCR demonstrated decreased expression of *IL37* and *LOR* in the ABCA12 KO model that was upregulated by 1400W treatment (Supplemental Figure 3, D and F).

### Tofacitinib markedly improved lipid secretion and barrier formation in the HI 3D model.

**Figure 7:**
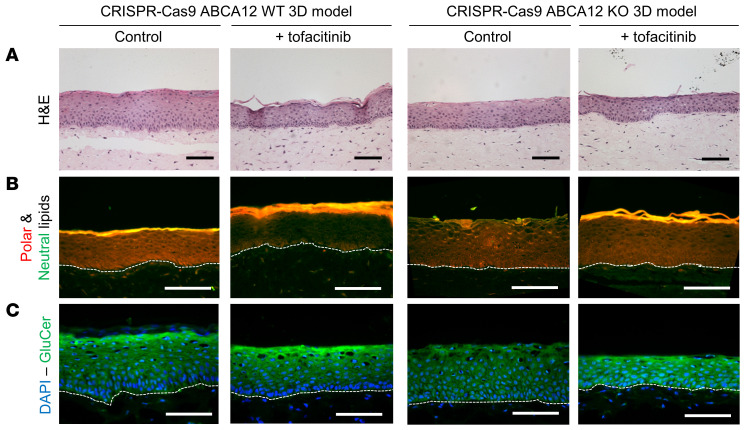
Tofacitinib treatment improved skin-barrier formation in the WT and HI 3D model. Representative (**A**) H&E (bright-field channel), (**B**) polar/neutral (red/green channel), (**C**) GluCer (green channel), and DAPI (blue channel) staining images of in vitro WT and HI 3D models with or without tofacitinib from 3 independent biological replicates. Scale bars: 100 μm.

To further investigate the increase of IFN-γ signaling in HI patient skin found in the RNA-Seq data set (Figure 3), we treated the WT and HI 3D models with the potent JAK inhibitor tofacitinib. Although p-JAKs were not detectable after 14 days of air-liquid interface growth (data not shown) and p-STAT1 activation was not observed in the HI model (Figure 4D), tofacitinib markedly improved the abnormal epidermal differentiation phenotype of the HI model with the appearance of terminally differentiated cells, as observed by H&E staining (Figure 7A). Tofacitinib treatment also strikingly improved the formation of the lipid barrier with restoration of polar and neutral lipid expression as well as GluCer in the stratum corneum–like layer, as detected by Nile red staining and GluCer staining, respectively (Figure 7, B and C). An increase in keratinocyte differentiation and lipid barrier formation was also observed in the in vitro WT 3D model (Figure 7, A–C).

The mRNA levels of *IL37* and *LOR*, downregulated in the HI 3D model compared with WT control, were upregulated by tofacitinib treatment (Supplemental Figure 3, E and G).

## Discussion

Deleterious mutations in *ABCA12*, a key component in skin barrier formation, underlie HI (4, 5). The lipid transport deficiency arising from those mutations results in the impairment of GluCer and other lipid loading in LBs, essential for the formation of the lipid barrier. In the ABCA12 KO cell line, a congested lipid droplet pattern was identified, recapitulating the observations from HI patient–derived cells, which were restored to a normal diffuse lipid pattern after ABCA12 gene correction (5).

Despite this discovery and the development and analysis of mouse models (6, 8–10) and in vitro 3D models (14) improving the understanding of the disease, no targeted and effective therapies have been developed.

The development of an in vitro HI 3D model, using an in-house–engineered CRISPR/Cas9 ABCA12 KO keratinocyte cell line, gave us insight into ABCA12 biology. We believe this human 3D model with complete loss of ABCA12 function mimics the most severe HI patient phenotype with homozygous loss-of-function mutations. The recapitulation of the HI histological skin characteristics, including dysregulation of epidermal differentiation (3), lipid expression, and loss of GluCer in the upper epidermis (18), validated the relevance of our model. However the HI 3D model does not exhibit hyperkeratosis, a hallmark of HI (4). We hypothesized that the impaired and extended differentiation of keratinocytes (large cells, some still nucleated, expressing involucrin) seen after 2 weeks growth mimics the early stage of disease development. The HI 3D model replicates well the basal/suprabasal epidermis of HI patient skin with early differentiation and enucleated cells in the spinous layer. If the in vitro model had a longer life span, one could predict a hyperkeratotic phenotype over time with absence of desquamation (14), as seen in HI.

The use of RNA-Seq technology offered us an unbiased and sensitive method for investigating the transcriptome of HI skin patient samples and ABCA12 KO–stratified keratinocytes. We identified genes significantly differentially expressed, clustering into 2 groups overlapping between the 2 data sets: epidermal development (epidermal keratinization and lipid metabolism) and inflammation (innate immunity and IFN-γ signaling). This confirmed transcriptome similarities between HI skin and the HI 3D model and thus the applicability of the HI 3D in vitro model. The study of a broad panel of inflammatory mediators also confirmed the proinflammatory secretome of ABCA12 KO cells. These findings are in keeping with the clinical findings in HI patients: highly inflamed, thickened skin (19).

A potential key role for the JAK/STAT signaling pathway in HI was revealed with the discovery of downregulation of the STAT inhibitors SOCS1 and SOCS3 and upregulation of the transcription factor STAT1 and related downstream targets in HI skin and in the vitro model. The JAK/STAT signaling pathway plays a central role in proliferation, cell death, inflammation, and angiogenesis through modulation of gene expression (20, 21). The literature reports that IFN-γ, the STAT1-signaling activating cytokine, is significantly increased in vivo in ARCI skin patients (LI and NBCIE patients, study not including HI patients) (13).

Activation of STAT1 was also identified in other skin diseases, such as systemic lupus erythematosus (22) and psoriasis (23). The JAK/STAT pathway is involved in many biological processes, including skin homeostasis (24). Upon activation, the transcription factor STAT1 can induce the transcription of its target gene *NOS2* (25), responsible for the increase of the free radical NO, a molecule having either beneficial or harmful effects on skin homeostasis depending on its concentration and length of exposure (26). Also, increased levels of intracellular ceramides can upregulate NOS2 (27). A high concentration of NO is proinflammatory and inhibits epidermal barrier formation (28, 29), which correlates with the HI 3D model and patient skin phenotype, but also with the phenotypic changes observed in the WT 3D model after treatment with the NO-releasing agent SNAP. To investigate whether NO could be involved in the pathophysiology of HI, we looked at NOS2 expression levels and found substantial upregulation in the HI 3D model and patient skin. Despite CRISPR/Cas9 ABCA12 KO cells being more responsive to IFN-γ treatment, we did not detect positive p-STAT1 nuclei in the in vitro IFN-γ unstimulated HI 3D model, suggesting that other transcription factors, such as NF-κB, C/EBP, or AP-1, also play a role, at least in vitro, in regulating *NOS2* transcription (30). We detected an increase in FOSL2, the AP-1 subunit, in the HI 3D model. Increased NOS2 results in high, long-lasting levels of NO (31), involved in many cutaneous physiological processes, including vasodilatation, apoptosis, inflammation, wound healing, and epidermal differentiation (26).

Upregulation of the STAT1/NOS2 pathway has not been reported in HI before; however, it has been described in other inflammatory skin diseases, such as psoriasis and atopic dermatitis (26, 32), sharing characteristics of skin barrier impairment and increased inflammation. It is interesting to note that tofacitinib, a potent inhibitor of the JAK family that has significantly improved the HI 3D phenotype, is already licensed for treating active psoriatic arthritis and rheumatoid arthritis (33), revealing the pertinence of targeting the JAK/STAT pathway in inflammatory skin diseases, such as HI. In moderate-to-severe psoriasis patients treated with tofacitinib, a small increase in serum lipid levels was reported (34), correlating with our observations in the HI in vitro model. Tofacitinib increased cornification and lipid secretion in both WT and HI 3D models, although p-JAKs were not detectable. This might suggest that either JAKs are transiently activated during 3D growth or the observed tofacitinib effect in our in vitro model is off target. Multiple off-target effects of tofacitinib have been reported previously (35). Additionally, the HI patient skin RNA-Seq data revealed upregulation of IL-17, a cytokine produced by Th17 immune cells, which is also an inducer of NOS2 expression, acting through STAT1, AP-1, and NF-κB transcription factors (36–38). Interestingly the use of the anti–IL-17 antibody secukinamab, is currently under investigation in ichthyosis patients (ClinicalTrials.gov NCT03041038) who express an elevated level of IL-17 and related cytokines (cohort not including HI patients) (13).

We also identified an increase of the innate immune cytokine IL-1α in the HI 3D model and IL-36α and IL-36γ, both IL-1 like cytokines, in HI patient skin. Both cytoplasmic and nuclear IL-36 expression were observed; the role of IL-36 in the nucleus, also reported in other studies (39, 40), is poorly understood. Also, a striking decrease of the inhibitor of immune response IL-37 was observed in the HI 3D model and patient skin (mRNA and protein), predicting a reduction of the antiinflammatory response. It was shown that IL-37 downregulates IL-17 and IL-17–related cytokines (e.g., IL-36) (41). Conversely, a decrease of IL-37 expression, intrinsic to ABCA12-deficient keratinocytes, might be involved in HI pathogenesis by favoring production of the IL-17 and downstream IL-36 cytokine family. This IL-1 family dysregulation has never been reported in HI before, but an IL-17 immunophenotype was recently identified in major orphan forms of ichthyosis (13) as well as psoriasis (42). In psoriasis, even if IL-36 biology remains to be fully investigated, its inhibition is believed to be a promising treatment, with a number of drugs under investigation in severe forms of psoriasis (e.g., generalized pustular psoriasis, palmoplantar pustular psoriasis) (43). In the current pathogenic model of psoriasis, IL-36 is believed to be a downstream event of IL-17 expression. Thus, the absence of IL-36 expression in the HI 3D model might be because of the lack of IL-17–expressing cells in our model (44). In the HI 3D model, the proinflammatory environment was confirmed with the increased proliferation of THP-1 immune cells. It has been reported in the in vivo mouse HI model that blockade of fetal inflammation with the use of the broad spectrum inflammation suppressor IL-37b leads to improvement of HI keratinocyte differentiation (12). Moreover, inhibition of IL-1 biological activity with use of an IL-1 receptor antagonist in a rat LI in vitro model also resulted in an improved phenotypic outcome with reduction of hyperkeratosis (45). The epidermal phenotype improvement with antiinflammatory treatments reported in these studies suggests to us that reducing skin inflammation would be beneficial for HI patients. Therapeutic strategies targeting the upregulated IL-36 and IL-17 as well as the overarching IL-23 pathway could be of benefit in the treatment of HI, as already shown in psoriasis (46).

To test whether targeting NOS2 is relevant for HI patient treatment, we used the potent, selective NOS2 inhibitor 1400W (47) in our HI 3D model. Striking recovery of the lipid barrier was observed. Also, the inhibition of NOS2 and consequently a decrease in the proinflammatory mediator NO would dramatically decrease HI skin inflammation. The NOS2 inhibitor 1400W that we used was a preclinical tool and did not progress to clinical development. However, other selective and potent NOS2 inhibitor compounds, such as GW274150 (48), which was used in completed several phase 2 clinical studies in migraine, asthma, and rheumatoid arthritis (DrugBank DB12237), could represent clinical candidates for HI treatment.

Overall, these findings reveal the potential for NOS2 inhibitors or JAK/STAT inhibitors, targeting the pathway upstream of NOS2, as HI therapeutic agents.

In summary, our study reveals the potential role of STAT1 and its downstream target NOS2 in the pathogenesis of HI and shows that blocking NOS2 reverses the permeability and lipid barrier impairment seen in our in vitro HI 3D model. These findings, together with the literature, strongly suggest that drugs targeting either NOS2 or the JAK/STAT1 pathway would be beneficial for treating HI patients and improving their quality of life. We also found major IL-1 family cytokine dysregulation, which could represent a further therapeutic target in HI patients.

## Methods

### Patient samples.

Normal skin was obtained from redundant surgery and HI skin from 4 adult HI patients after informed consent. The HI patient mutation details and therapy are shown in Supplemental Figure 4A. Materials from all 4 HI patients were used for RNA-Seq analysis (*n* = 4). For all the follow-up staining analysis, skin samples from patients HI 1, 2, and 3 were used (*n* = 3).

### RNA-Seq analysis.

Total RNA was isolated from ABCA12 WT and KO 3D models, skin biopsies from 4 HI patients, and 5 normal skin biopsies following the RNeasy Lipid Tissue Mini Kit (QIAGEN) manufacturer’s instructions. Extracted RNA was provided to the Barts and the London Genome Center (Queen Mary, University of London, London, United Kingdom). RNA samples were assessed for quantity and integrity using the NanoDrop 8000 spectrophotometer, version 2.0 (Thermo Scientific), and Agilent 2100 Bioanalyzer (Agilent Technologies), respectively. Samples displayed low levels of degradation, with RNA integrity numbers (RIN) between 6.7 and 10, and 100 ng of total RNA from each sample was used to prepare total RNA libraries using the NEBNext mRNA Kit in combination with the NEBNext Ultra Directional RNA Library Preparation Kit. Fragmentation before first-strand cDNA synthesis was carried out using incubation conditions recommended by the manufacturer for samples with an RIN of greater than 7 (94°C for 15 minutes), and 13 cycles of PCR were performed for final library enrichment. The resulting libraries were quantified using the Qubit 2.0 spectrophotometer (Life Technologies) and average fragment size assessed using the Agilent 2200 TapeStation (Agilent Technologies). Sample libraries were combined in equimolar amounts into a single pool. The final library pool was loaded to the NextSeq500 at 1.2 pM, and 75 bp paired-end reads were generated for each library using the Illumina NextSeq500 High-Output Kit. The raw FASTQ data were merged and mapped using Hisat2, version 2.0.4, in paired-end mode against version 38 of the human genome (GRCh38 soft-masked) obtained from ensembl 93 and splice sites obtained from ensembl GTF annotation, version 93: Homo_sapiens.GRCh38.93.gtf. They were converted into sorted BAM files using samtools view –bS. BAM files were quantitated against gene expression at the gene level using htseq-count, version 0.6.0, against version 93 of the Ensembl Human GTF file Homo_sapiens.GRCh38.93.gtf. These count files were loaded into R/BioConductor using the DESeq2 package function DESeqDataSetFromHTSeqCount. The differentially expressed genes abs(log_2_(fold change)) > 1 and –log_10_ *P_adj_* > 2 for the HI skin data set or greater than 1.3 for the HI 3D model were represented on a volcano plot using PRISM software (GraphPad), and hits were visualized on a heatmap generated with RStudio software. The top relevant enriched GO terms identified using DAVID online tool (https://david.ncifcrf.gov) and the corresponding genes were noted and transcribed in the results. We used the software STRING (https://string-db.org) to create functional protein association networks to visualize the protein-protein interaction relationship. To estimate the changes in immune infiltrates in HI skin compared with normal skin using RNA-Seq gene expression data, we used CIBERSORT (https://cibersort.stanford.edu) with the provided LM22 signature genes file (22 immune cell types) including both relative and absolute modes. For RNA-Seq data, the quantile normalization setting was disabled, as suggested by the authors (49). All original RNA-Seq data sets were deposited in the NCBI’s Gene Expression Omnibus (GEO GSE131903).

### N/TERT, THP-1, and human primary fibroblast cell culture.

The human keratinocyte telomerase reverse-transcriptase–immortalized (h/TERT-immortalized) N/TERT-1 cell line derived from clinically normal foreskin tissue and supplied by James Rheinwald (Department of Dermatology, Harvard University Medical School, Boston, Massachusetts, USA) (50) was grown in RM^+^ growth media (DMEM/F-12, 10% FBS, 1× penicillin streptomycin [P/S], 0.4 μg/mL hydrocortisone, 0.5 μg/mL insulin, 10 ng/mL epidermal growth factor, 0.1 nM cholera toxin, 5 μg/mL transferrin, 20 pM liothyronine) and incubated at 37°C, 5% CO_2_. Human primary fibroblasts isolated from fresh redundant skin were grown in fibroblast growth media (DMEM, 10% FBS, 1× P/S) and incubated at 37°C, 5% CO_2_. The THP-1 suspension cell line (ATCC TIB-202) was grown in THP-1 growth media (RPMI with 0.05 mM 2-mercaptoethanol, 10% FBS, 1× P/S) and incubated at 37°C, 5% CO_2_. The HPV-16–immortalized HI patient–derived cell line (HI) (generated by Andrew South’s laboratory, School of Medicine, University of Dundee, United Kingdom, now at Thomas Jefferson University, Philadelphia, Pennsylvania, USA) and the normal skin cell line (WT) (immortalized in the same way generated in-house) were grown in keratinocyte low-calcium growth media (Epilife, Human Keratinocyte Growth Supplement [HKGS], Thermo Fischer Scientific), 1× P/S) and incubated at 37°C, 5% CO_2_. All cell lines were mycoplasma negative.

### CRISPR/Cas9 KO of ABCA12.

The Dharmacon Edit-R CRISPR/Cas9 platform was used to generate the CRISPR/Cas9 ABCA12 KO cell line. The crRNA, sequence: 5′-GCTGCCATACCGTATGTCCCTGG-3′, was designed to target exon 27 of the *ABCA12* gene, and 25% confluent N/TERT keratinocytes were transfected with mKate Cas9 plasmid (1 μg/mL final, Dharmacon) using FuGENE 6 transfection solution (3 μL/mL final, Promega) diluted in RM^+^ growth media, antibiotic free. After overnight incubation (37°C and 5% CO_2_), cells were trypsinized and fluorescent mKate cells were selected using a BD Bioscience Aria IIIu sorter fitted with the 660/20 nm filter. Positive mKate cells were seeded back into 24-well plates in normal growing media at a density of 1.6 × 10^4^ cells/well and incubated overnight. The following day, the CRISPR/Cas9 transfection mix was prepared as follows: crRNA (50 nM final), trRNA (50 nM final, Dharmacon), and HiperFect transfecting reagent (7.5 μL/mL final, QIAGEN); this was diluted in RM^+^ growth media, added to the previously sorted cells, and incubated overnight (37°C, 5% CO_2_). The day after the transfection, media were replaced with RM^+^ growth media and cells were left to grow to confluence. When confluent, cells were single cell sorted into 96-well plates and growing clones were then harvested to be screened. For the CRISPR/Cas9 WT cell line, the same protocol was applied, but no crRNA was used. Genomic DNA extraction was performed using the DNeasy Tissue Kit (QIAGEN), and ABCA12 exon 27 was amplified by PCR using the primers forward, 5′-TGGAAACTGAGACCACCTTTT-3′, and reverse, 5′-GAGTCCAAAGACGCATGTGTAG-3′, and the Reddymix PCR Master Mix (Thermo Fisher Scientific). Sanger sequencing of exon 27 was performed, and electropherograms were visualized with Chromas software (Technelysium). The WT (NM_173076.2) and mutant (AC deletion) mRNA sequences were translated using the ExPaSy online tool (SIB), and protein sequences were aligned using Clustal Omega software (EBI). In silico analysis was carried out using the Off-Spotter tool (https://cm.jefferson.edu/off-spotter-code) to identify possible off-target sequences. No off-target genomic sites with fewer than 5 mismatches were identified.

### IFN-γ treatment.

Four days after seeding, 2D cultured cells were treated with IFN-γ at 2.5 ng/mL for 30 minutes before harvesting for immunoblotting analysis.

### Growth curve and cell morphology analyses.

Cells were cultured, and at given time points (24 to 96 hours), cells were paraformaldehyde fixed, Triton permeabilized, stained with DAPI and CellMask (H32712, Invitrogen), and imaged using the INCell Analyzer 2200 (GE); data were analyzed with the INCell analysis software.

### Cytokine measurement.

Secreted cytokine levels, normalized to the total cell number, were measured in the supernatant of cell lines after 4 days growth, using the human cytokine array (R&D Systems, ARY005B) and human quantitative IL-1α and CXCL1 ELISA kits (R&D Systems, DY200 and DY275) according to the manufacturer’s instructions.

### 3D model generation.

In vitro 3D models were generated by placing 340 μL of collagen/Matrigel matrix containing 3.4 × 10^4^ fibroblasts (5 volumes of collagen I, 2 volumes of Matrigel, 1 volume of 10× MEM, 1 volume of FBS, and 1 volume of primary fibroblasts, resuspended at 1 × 10^6^ cells/mL in fibroblast growth media) into a 12-well plate Transwell insert (Millipore) and incubating for 1.5 hours at 37°C. After polymerization of the dermis-like layer, 3.4 × 10^5^ N/TERT cells (resuspended at 1 × 10^6^ cells/mL in RM^+^ growth media) were seeded on top of the matrix into a cloning ring and RM^+^ growth media were added underneath the insert. After overnight incubation at 37°C, the cloning rings were removed, and the 3D models were air lifted and left to grow at an air/liquid interface for 14 days with daily media changes. The RM^+^ media were used for the first 8 days, and then RM^+^ media supplemented with 50 μg/mL l-ascorbic acid (MilliporeSigma) was used for the rest of the culture. At the end time point, the 3D models were either harvested for NO measurement or cut in half: one half was fixed in 4% PFA for 30 minutes at RT and then embedded in paraffin, and the other half was frozen in OCT. 5 μm paraffin or frozen sections were generated with a microtome and stored appropriately. When THP-1 cells were incorporated in the dermis-like layer, the same protocol was used with the addition in the dermis-like layer of 1 volume of THP-1 cells resuspended at 1 × 10^6^ cells/mL in THP-1 growth media. The THP-1 cell area was determined using CellProfiler image analysis software (Broad Institute).

### NOS2 inhibitor, tofacitinib, and SNAP treatment.

Daily treatment used 1 μM of the selective and potent NOS2 inhibitor 1400W (47) (1415, Tocris), 100 nM of the JAK1/3 inhibitor tofacitinib (35) (sc-364726, Insight Biotechnology), or 50 μM of the NO-releasing agent S-Nitroso-*N*-acetyl-DL-penicillamine (SNAP) (51) (sc-200319, Santa Cruz Biotechnology), which was added to the 3D models for 8 days.

### Permeability assay analysis.

The 3D models were incubated for 4 hours with 1 mM Lucifer yellow (L0259, MilliporeSigma) solution on their upper surface, then PBS washed and cryoembedded. Images were captured using an Epifluorescence Leica microscope.

### Intracellular NO measurement.

Total NO (nitrate and nitrite) production of 3D models was measured following the manufacturer’s protocol (ab65327, Abcam).

### qPCR analyses.

qPCR was performed using the following primers: *ABCA12*, forward, 5′-AATACGATGCTGCCCGACAT-3′, reverse, 5′-ACTGCTGATTGTGGCTTGTTC-3′; *NOS2*, forward,5′-TGGTGCTGTATTTCCTTACGAGGCGAAGAAGG-3′, reverse, 5′-GGTGCTACTTGTTAGGAGGTCAAGTAAAGGGC-3′; *SOCS1*, forward, 5′-TCCGATTACCGGCGCATCACG-3′, reverse, 5′-CTCCAGCAGCTCGAAAAGGCA-3′; *SOCS3*, forward, 5′-GGAGTTCCTGGACCAGTACG-3′, reverse, 5′-TTCTTGTGCTTGTGCCATGT-3′; *IL37*, forward, 5′-AGTGCTGCTTAGAAGACCCG-3′, reverse, 5′-CCCAGAGTCCAGGACCAGTA-3′; *HPRT*, forward, 5′-GAAGAGCTATTGTAATGACC-3′, reverse, 5′-GCGACCTTGACCATCTTTG-3′; *LOR*, forward, 5′-GCTTTGGGCTCTCCTTCCTT-3′; and reverse, 5′-AGGTCTTCACGCAGTCCAC-3′. Relative mRNA expression was determined using the 2^–ΔΔCt^ method (HPRT normalization).

### Western blot analyses.

Lysates of cultured keratinocytes were prepared using RIPA buffer (Thermo Fisher Scientific) and protease inhibitors (Roche). Total protein was quantified using the BCA assay (Thermo Fisher Scientific). 15 μg of total protein was resolved on 4%–12% SDS-PAGE gradient gels, then electrophoretically transferred to a nitrocellulose membrane. The following primary antibodies were used: STAT1 (1:1000 dilution, Cell Signaling Technology, catalog CST-9172P), p-STAT1 (1:1000 dilution, Cell Signaling Technology, clone D4A7, catalog CST-7649), FOSL2 (1:100 dilution, Santa Cruz Biotechnology Inc., clone G-5, catalog sc-166102), GAPDH (1:10,000 dilution, Abcam, catalog ab9485), and ABCA12 (1:500, Abcam, catalog ab98976). Protein bands were visualized using HRP-conjugated secondary antibodies and the ECL-Plus Detection Kit (GE Healthcare). Band intensities were measured using ImageJ (version 1.52a, NIH) and normalized against the corresponding endogenous GAPDH levels.

### Immunofluorescence and histological analyses.

Air-dried cryosections were paraformaldehyde fixed and stained with H&E and the following primary antibodies (dilution 1:100): GluCer (Glycobiotech, catalog RAS_0011), Involucrin (Abcam, catalog ab98), NOS2 (R&D Systems, catalog MAB9502-SP), ABCA12 (Abcam, catalog ab98976), p-STAT1 (Cell Signaling Technology, catalog CST-9167), STAT1 (Cell Signaling Technology, catalog CST-9172), IL-37 (BioTechne, catalog NBP2-33712), IL-36α (R&D Systems, catalog AF1078), and IL-36γ (Thermo Fisher Scientific, catalog MA526240). Quantification was performed using CellProfiler software.

### 2D culture and 3D model Nile red staining.

A drop of 2.5 μg/mL Nile red (479918, MilliporeSigma) was added to live cells or air-dried cryosections, which were immediately covered with a coverslip. Green and red channel images were taken using an Epifluorescence Leica microscope.

### Statistics.

Statistical analyses used unpaired, 2-tailed Student’s *t* test or 2-way ANOVA for multiple comparisons. Data represent the mean ± SD or ± SEM. Statistical analysis was performed using GraphPad Prism software (version 8 for Windows). The null hypothesis was rejected at a significance level of *P* < 0.05.

### Study approval.

This study was approved by the National Health Service Health Research Authority London – South East (REC reference 08/H1102/73) and London – City and East (REC reference: 05/Q0603/9) research ethics committees and was conducted according to Declaration of Helsinki principles.

## Author contributions

FE, MPC, and EMJ conducted experiments shown in the paper. PD conducted preliminary experiments. FE, MPC, and EAO designed and analyzed experiments. AJE performed bioinformatics analysis. MAM and DPK were involved in supervision of the work. FE and EAO wrote the manuscript with input from all the authors. EAO obtained funding and has overall responsibility for the study.

## Supplementary Material

Supplemental data

## Figures and Tables

**Table 1:**
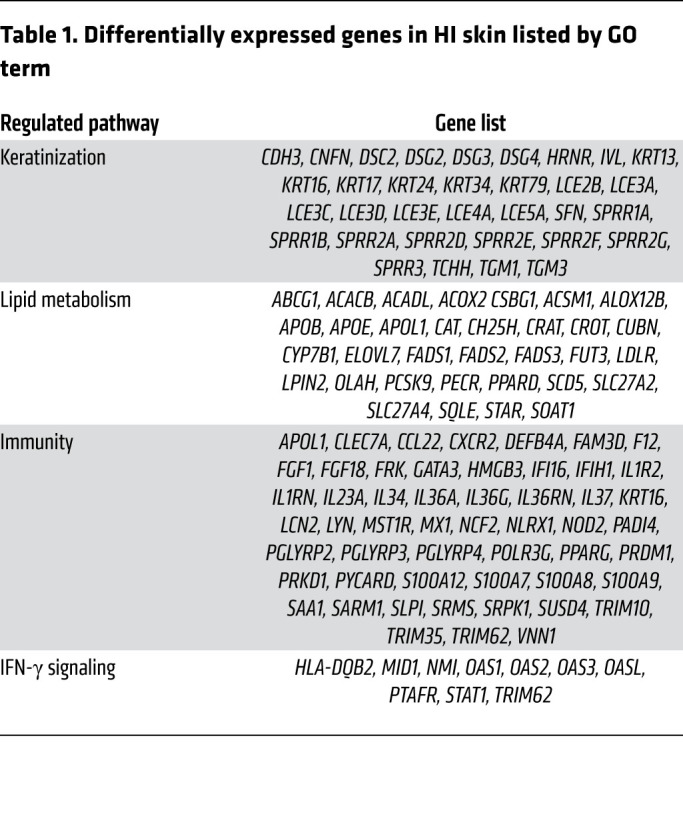
Differentially expressed genes in HI skin listed by GO term

**Table 2:**
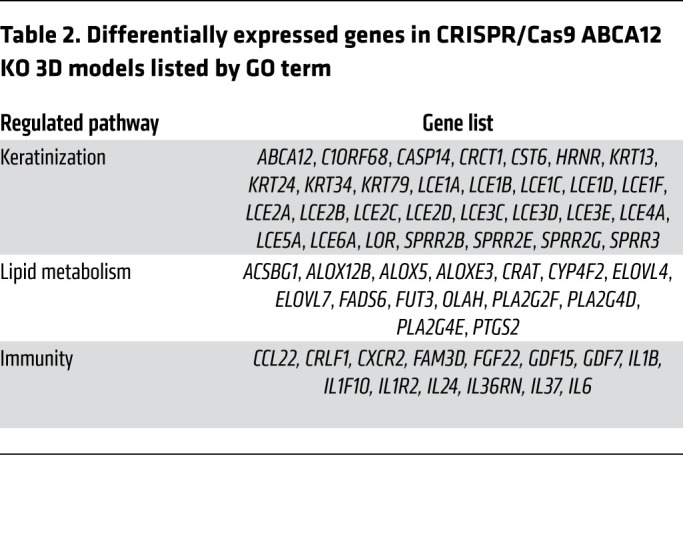
Differentially expressed genes in CRISPR/Cas9 ABCA12 KO 3D models listed by GO term

## Version 1 06/16/2020

In-Press Preview

## Version 2 08/10/2020

Electronic publication

## Version 3 09/01/2020

Print issue publication
