# Supplementary material for: 3D model of harlequin ichthyosis reveals inflammatory therapeutic targets
Source: J Clin Invest. 2020 Aug 10;130(9):4798–810. doi: 10.1172/JCI132987 (PMC7456239; doi:10.1172/JCI132987)
Supplement: Supplemental data [file jci-130-132987-s229.pdf]

Supplementary Figures

A B

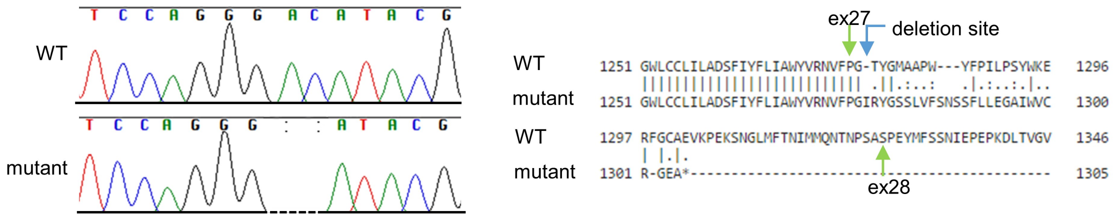

C

Gene Name	chromosome	strand	start	end	given query	actual genomic hit	No. of mismatch
RIN2	20	-	19937315	19937337	GCTGCCATACCGTATGTCCC	GCTGtCATAgCcTgaGTCCC-GGG<br>Rev Comp: GGGACtcAgGcTATGaCAGC	5
SAG	2	-	234217821	234217843	GCTGCCATACCGTATGTCCC	GCTGCCATgttaTcTGTCCC-TGG	5
DNAH8	6	-	38997921	38997943	GCTGCCATACCGTATGTCCC	ctTcCCATtCCGTcTGTCCC-AGG	5
DNAH8	6	+	38957938	38957960	GCTGCCATACCGTATGTCCC	GCTGatATcaCGTAaGTCCC-TGG	5
PAGR1	16	-	29827699	29827721	GCTGCCATACCGTATGTCCC	GCgGCCAgACCGcccGTCCC-CGG<br>Rev comp: GGGACgggCGGTcTGGCcGC	5

D E

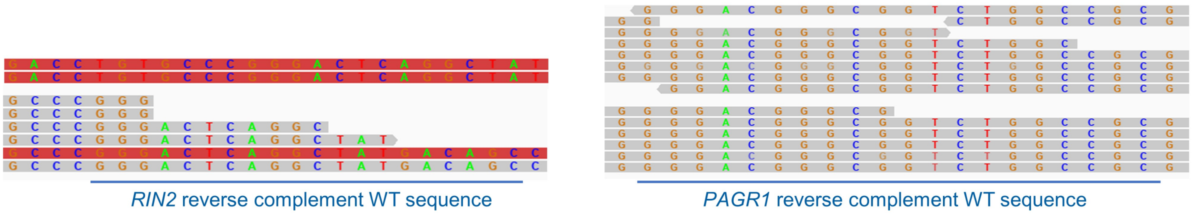

Figure S1 ABCA12 KO cell model generation and off-target analysis.

(A) Schematic representing electropherograms of the Sanger sequenced *ABCA12* exon 27 from gDNA of ABCA12 WT and KO cell lines. **(B)** Alignment of predicted ABCA12 protein of ABCA12 WT and KO. The deletion site is labelled with a blue arrow; exon 27 and exon 28 starting positions are labelled with a green arrow. In silico analysis was carried out using the Off-Spotter tool. **(C)** 5 potentials off target sequences were identified within coding regions with 5 mismatches (written in lower case). Analysis of RNA-Seq data from ABCA12 WT and KO cells identified that only *PAGR1* and *RIN2* are expressed in keratinocytes. No mutations were observed in **(D)** *RIN2* or in **(E)** *PAGR1*.

A B

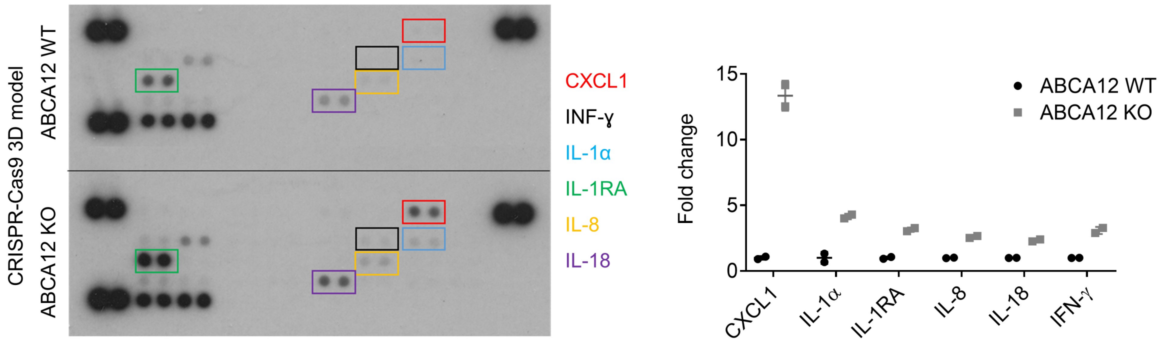

Figure S2 Cytokine array

Conditioned media from ABCA12 WT and KO cells was collected after 72h, cells were counted and media volume normalised to cell number. **(A)** Soluble cytokines were analysed using Human Cytokine Array (R&D Systems) and **(B)** relative amounts quantified using densitometry (each dot represents 2 technical replicates).

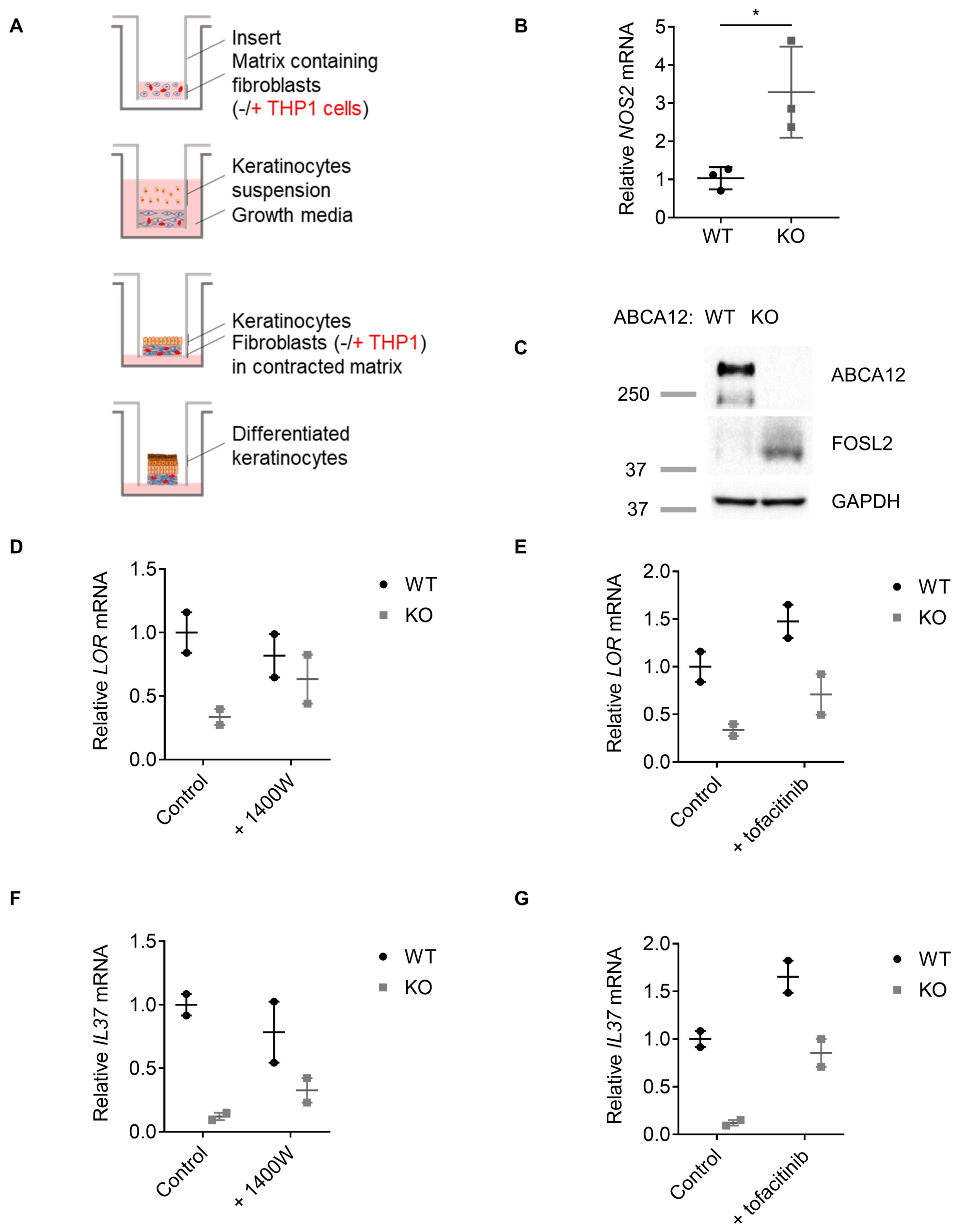

Figure S3 HI 3D model development and characterisation

(A) Schematic illustrating the steps to generate keratinocyte organotypic cultures in transwell insert, with and without the addition of THP-1 immune cells in the dermis-like layer. **(B)** Quantitative qPCR analysis of *NOS2* in ABCA12 WT and KO 3D model lysates (each dot represents the mean of 3 technical replicates, n=3, mean ± SD, unpaired t test, *: P ≤ 0.05). **(C)** Representative Immunoblot of ABCA12, FOSL2 and GAPDH proteins in ABCA12 WT and KO 3D model lysates. The GAPDH blot was run in parallel, contemporaneously, with the ABCA12 and FOSL2 blots. Quantitative qPCR analysis of **(D, E)** Loricrin (*LOR*) and **(F,G)** *IL37* in ABCA12 WT and KO 3D model lysates control or with 1400W or tofacitinib treatment respectively (each dot represents the mean of 3 technical replicates, n=2, mean ± SEM).

A

HI patient	*ABCA12* mutation	Oral treatment
HI 1	p.E675Rfs*14 (exon 16), p.R1297* (exon 27)	No
HI 2	p.C2440Ffs*24 (exon 49)	No
HI 3	p.E2264* (exon 45), c.5382-2a/g (intron 34) splice site	Acitretin
HI 4	p.R44G (exon 2), p.N678S (exon 16)	Acitretin

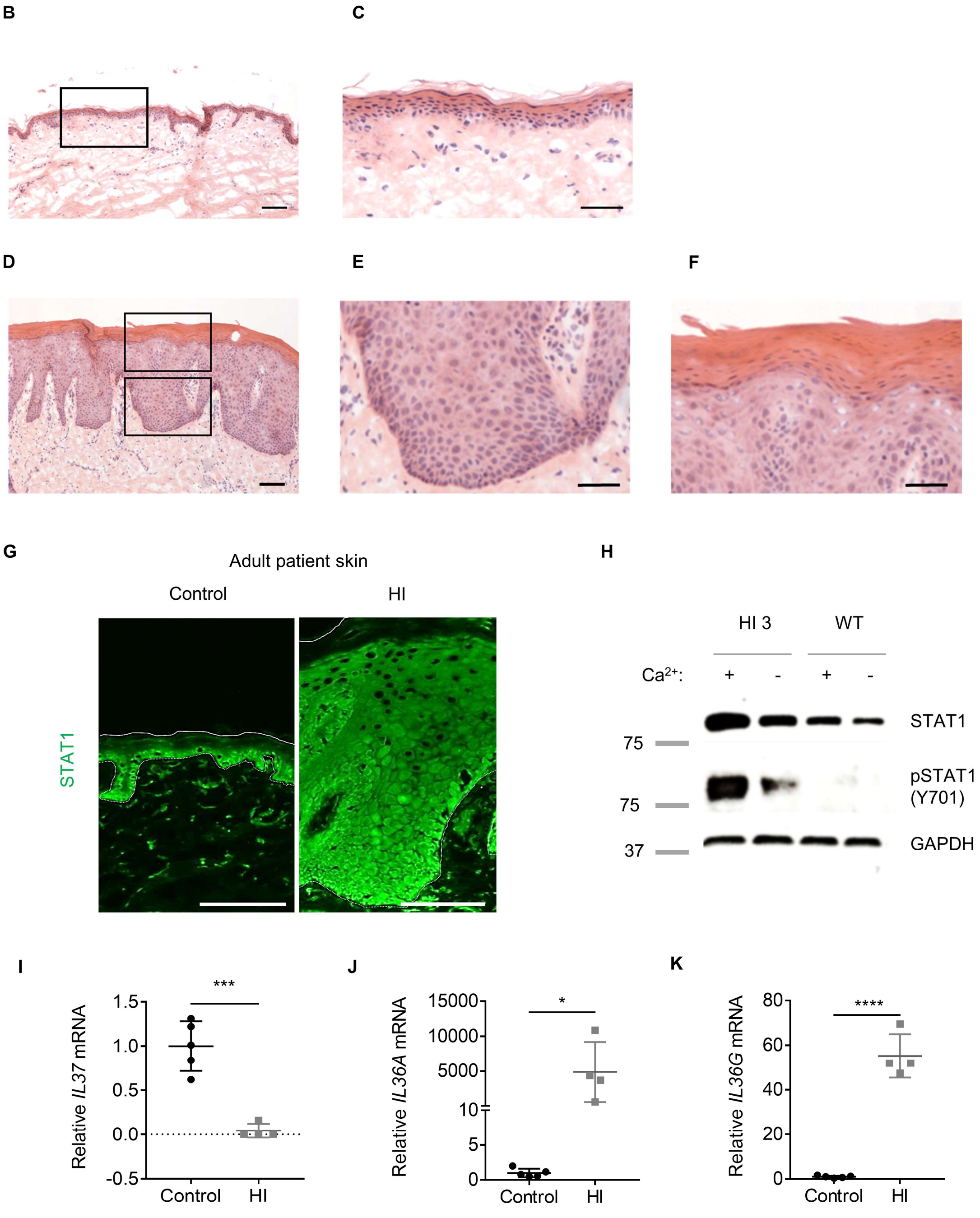

Figure S4 HI skin sample characterization

(A) Table summarizing the 4 HI patients *ABCA12* mutation status and the treatment details. Representative image of normal skin **(B)** low power, scale bar: 100 µm and **(C)** high power, scale bar: 50 µm. Representative image of HI skin **(D)** low power, scale bar: 100 µm and high power of **(E)** basal and suprabasal layers, **(F)** granular and corneal layers, scale bar: 50 µm. **(G)** Representative high power STAT1, scale bar: 100 µm. **(H)** Representative Immunoblot of pSTAT1 (Y701), total STAT1 and GAPDH proteins in WT and HI cell patient HPV-16 immortalised cell lysates. Quantitative qPCR analysis of **(I)** *IL37*, **(J)** *IL36A* and **(K)** *IL36G* (each dot represents the mean of 3 technical replicates, n=4 or 5, mean ± SD, unpaired t test, *: $P \leq 0.05$, ***: $P \leq 0.001$, ****: $P \leq 0.0001$).

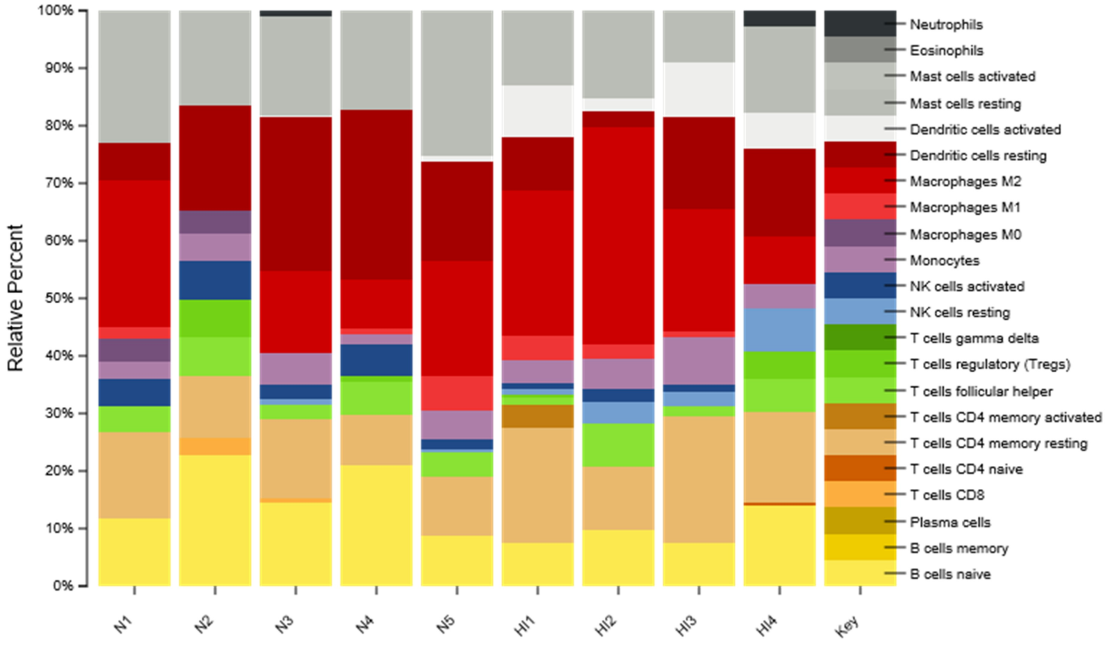

Figure S5 Immune cell types in HI skin versus normal skin

The software CIBERSORT was used to estimate the changes in immune infiltrates in HI skin (n=4) compared to normal skin (n=5) using the RNA-Seq gene expression data and the provided LM22 signature genes file (22 immune cell types) with both relative and absolute modes. The colour key on the right shows the different immune cell types.
